# Reading Disability and Quality of Life Based on Both Self- and Parent-Reports: Importance of Gender Differences

**DOI:** 10.3389/fpsyg.2016.01942

**Published:** 2016-12-15

**Authors:** Judit Balazs, Monika Miklosi, Krisztina T. Toro, Diana Nagy-Varga

**Affiliations:** ^1^Department of Developmental and Clinical Child Psychology, Institute of Psychology, Eötvös Loránd UniversityBudapest, Hungary; ^2^Vadaskert Child Psychiatry HospitalBudapest, Hungary; ^3^Heim Pál Pediatric Hospital, Budapest, Centre of Mental HealthBudapest, Hungary; ^4^School of Ph.D. Studies, Semmelweis UniversityBudapest, Hungary; ^5^Vecses City Local Government Department of Children and Family ServicesVecsés, Hungary

**Keywords:** reading disability, RD, dyslexia, quality of life, QoL, comorbidity

## Abstract

**Purpose:** The aim of this study is to investigate self- and parent-rated quality of life (QoL) in children with a reading disability (RD) and the impact of comorbid psychopathology, with special focus on age and gender differences.

**Methods:** Using the Dyslexia Differential Diagnosis Maastricht-Hungarian standard test, 127 children (aged < 18) were included in the RD group and 81 in the control group. To measure comorbid psychopathology, the Strengths and Difficulties Questionnaire (SDQ) was administered. To evaluate the children's QoL self- and parent-rated versions of the Measure of Quality of Life for Children and Adolescents (ILK) were used. Group differences in QoL and psychopathology were assessed using Mann-Whitney *U*-tests. Moderated mediational models were tested in which comorbid psychopathology mediated the relationship between group membership and self- and parent-rated QoL, which was dependent on gender. Child's age and parents' level of education were included as covariates.

**Results:** The RD group showed lower QoL than the controls in several domains, according to the parent-report, while no differences between the two groups were found, according to self-report. In boys, results revealed conditional and indirect effects of group membership on self- and parent-rated QoL through comorbid psychopathology (−0.046, BCa 95% CI: −0.135 to 0.043 and 0.064, BCa 95% CI: 0.024–0.111, respectively) as well as a conditional direct effect of group membership on parent-reported (−0.098, BCa 95% CI: 0.012–0.184), but not self-rated, QoL. No relationship was found for girls.

**Conclusions:** This study highlights the importance of measuring QoL and comorbid psychopathology in children with RDs from more sources and accounting for gender and age differences.

## Introduction

Reading disability (RD) belongs under the heading, Specific Learning Disorders (SpLD), in the classification group, Neurodevelopmental Disorders, in the Diagnostic and Statistical Manual of Mental Disorders, 5th edition (DSM-5) (American Psychiatric Association, 2013). It manifests in childhood and is not caused by impairments in general intelligence or in hearing and vision, inadequate schooling, or motivation and poor socio-cultural status (Karande and Venkataraman, 2012; American Psychiatric Association, 2013).

In the classroom, children with RD face several difficulties in their academic performance due to this impairment, which can cause anxiety, low self-esteem, behavior problems, and difficulties in social interactions (Alexander-Passe, 2006; Mugnaini et al., 2009; Kempe et al., 2011). Several studies have described the high comorbidity of RD, with both externalizing and internalizing disorders (Carroll et al., 2005; Germanò et al., 2010; Kempe et al., 2011). Among all comorbidities, attention deficit-hyperactivity disorder (ADHD) occurs most often with RD: 15–40% of children with RD have ADHD as well (Carroll et al., 2005; Germanò et al., 2010; Boada et al., 2012; Levy et al., 2013). Among internalizing disorders, anxiety is the one most often comorbid with RD—in 9–25% of cases (Riddick et al., 1999; Carroll et al., 2005; Goldston et al., 2007; Whitehouse et al., 2009).

Although the construct of the quality of life (QoL)—which includes psychical, psychological, and social aspects—has been recently gaining importance as a multidimensional measure of overall well-being of children with psychiatric problems (Bastiaansen et al., 2004, 2005; Dallos et al., 2014), there is very little research on the QoL in children with RD (Karande et al., 2009; Rotsika et al., 2011; Karande and Venkataraman, 2012, 2013; Ginieri-Coccossis et al., 2013). All these studies examined children with SpLD and not only children with RD. These data show that several aspects (e.g., emotional well-being, self-esteem and satisfaction in their relationships with family and friends) of self-perceived and parent-reported QoL for children with newly-diagnosed SpLD are poorer than the QoL of typically developed children (Karande et al., 2009; Rotsika et al., 2011; Karande and Venkataraman, 2012, 2013; Ginieri-Coccossis et al., 2013).

Although several researcher highlight that next to children's self-reported QoL, parents' views about their children's QoL should be measured, there is discrepancy between the agreement of parent-child proxy reports on children's QoL in the majority of the studies (Bastiaansen et al., 2004; Britto et al., 2004; Chang and Yeh, 2005; Cremeens et al., 2006; Russell et al., 2006; Eiser and Jenney, 2007; Jozefiak et al., 2008; Upton et al., 2008; Danckaerts et al., 2009; Eiser and Varni, 2013; Dallos et al., 2014), including the very few ones on children with SpLD (Rotsika et al., 2011; Karande and Venkataraman, 2013). Comparing the QoL of children with SpLD and typically developed peers (Rotsika et al., 2011), found significant differences between the parent-child proxy ratings: mothers of children with SpLD reported significantly lower scores regarding the child's functioning in school, but significantly higher scores in their physical and emotional well-being. However, between typically developed children and their mothers' proxy ratings, significant differences were found in physical well-being and self-esteem; in both cases parents reported higher scores.

There are only limited and controversial data on the impact of comorbidity on the QoL of children with RD (Karande and Bhosrekar, 2009; Karande and Venkataraman, 2013). Karande and Bhosrekar (2009) reported that parent-rated QoL of children with SpLD and comorbid ADHD was significantly poorer than for children with SpLD but without comorbid ADHD. In contrast Karande and Venkataraman (2013) found that children with SpLD and comorbid ADHD perceive their physical and psychosocial functioning better than children with SpLD but without comorbid ADHD.

Regarding gender and age differences in QoL, previous population studies show that children have better QoL than adolescents; moreover, there is no gender differences in QoL at young ages; however, girls score their QoL poorer than boys as their ages increase (Bisegger et al., 2005; Cavallo et al., 2006; Torsheim et al., 2006; Michel et al., 2009). In a clinical sample of children and adolescents who were referred to outpatient psychiatric clinics, the impact of psychopathology on QoL was larger for girls than for boys and the impact of psychopathology on QoL increased with increasing age (Bastiaansen et al., 2005).

The recognition and response to SpLD, including stigma, are different in different cultures, which may be associated with the level of support provided for children with SpLD. These factors influence the QoL of those suffering from SpLD. In Hungary, where this study was conducted, the level of stigma around SpLD is generally low and there are state sponsored programs to provide special (“developmental”) education for children suffering from SpLD. Furthermore, if needed, these children can get easier tasks in the school and extra time at the exams.

The aim of the current study was to investigate both the self-perceived and the parent-rated QoL of children with RD and the impact of comorbid psychopathology, with special focus on age and gender differences.

Our hypotheses were:

1. The QoL of children in both RD and healthy control groups decreases with increasing age according to both self- and parent-reports.
2. Higher level of psychopathology is associated with lower QoL in both RD and healthy control groups and in both boys and girls together, again, based on both self- and parent-reports.
3. According to parent reports, the QoL of children with RD is significantly lower than the QoL of healthy children in both boys and girls, however there is no differences between the QoL of children with RD and controls in both boys and girls according to self-reports.
4. The group of children with RD shows significantly high level of psychopathology than the healthy control group, both in boys and girls, based on both self- and parent-reports.
5. Comorbid psychopathology mediates between RD and both self- and parent-rated QoL, both in boys and girls.

## Methods

### Participants

The study was approved by the Ethical Committee of the Ministry of Human Capacities, Hungary. The parents of each child and children older than 14 years included into this study provided written informed consent after being informed of the nature of the study. Children under 14 years received both written and oral information about the project. In Hungary according to the low (Civil code 12. §) people above the age of 18 have legal capacity, children above 14 years have limited legal capacity (Civil code 12.A. §) and children below 14 are incapable (Civil code 12.B. §).

Study participants were recruited from Vecsés City Local Government Department of Children and Family Services, Vecsés, Hungary. Inclusion criteria were children had to be <18 years and been diagnosed with an RD, according to a computer-based dyslexia test (See Measures below). A control population from the same age group was recruited from the local community through word of mouth; here, the inclusion criterion was not having an RD, according to the dyslexia test. For both study groups, the exclusion criterion was having had a diagnosis of mental retardation in the medical history. Additionally, in the control group, exclusion criterion was any current or previous psychological or psychiatric treatment in the medical history.

### Measures

#### Dyslexia differential diagnosis Maastricht-Hungarian standard test (3 DM-H)

To verify RD, we used the shortened version of the 3 DM-H test (Tóth et al., 2014). The series of instructions, the presentation of stimuli and the participants' responses as well as the test's evaluation is computerized. The computer records the speed and the accuracy of the answer. Beyond the reading and orthographic capacity, the exercises also measure the most important reading-orthographic indicators. Additionally, the test includes the capacity of short-term memory, and it measures the simple, choice reaction time. Children who scored below the standard deviation (*SD* = −1; *T* = 40 in the 3 DM-H scoring system) on the main indexes of the battery (Cognitive Reading and Spelling Index) and on at least two main indicators (Reading scale, Spelling scale, Phoneme Awareness scale, Letter-Sound Correspondence scale, and the Rapid Automatized Naming scale) were considered dyslexic and formed the RD group.

#### Strengths and difficulties questionnaire (SDQ)

Psychopathology was evaluated using the Hungarian version of the SDQ (Goodman et al., 1998; Birkás et al., 2007; Turi et al., 2011, 2012). In this study, we used the parent-reported version of the scale. SDQ is a brief instrument for screening childhood behaviors. It consists of 25 items, which are distributed across five scales of five items each: prosocial behavior, hyperactivity-inattention, emotional symptoms, conduct problems, and the peer relationship problems scales. All the scales together, except the prosocial scale, form the SDQ Difficulties scale. The total difficulties score is generated by summing scores of the difficulties scales. In this study we used this score. Each item could be answered as “not true,” “somewhat true,” and “certainly true.” High scores indicate high levels of psychopathology. In the present sample, internal consistency of the SDQ total difficulties score was acceptable (α = 0.62).

#### Erfassung der lebensqualität kindern und jugendlichen (measure of quality of life for children and adolescents) (ILK)

QoL was measured by the Hungarian version of the ILK scale (Mattejat and Remschmidt, 1998; Kiss et al., 2007). The ILK scale evaluated six domains of QoL: school, family, peer relations, time spent alone, somatic and mental health, and general QoL using seven items. Both self-report (child version: participants <12 years old; adolescent version: participants ≥12 years old) and the parent version of ILK were used. The adolescent and the parent versions of the ILK used a 5-point Likert scale (1 = *less impairment*, 5 = *the most serious impairment*), while the child version of the scale used faces expressing emotions (laughing, smiling, neutral, sad, and crying) for the evaluation of the items. High scores indicate low levels of QoL. Reliabilities of the self-rated and parent report versions of the measure in the present sample were good to very good (α = 0.76 and 0.85, respectively).

### Statistics

In a bivariate analysis, we explored group differences in self- and parent-reported QoL and psychopathology in the total sample, separately for girls and boys, and their associations with age. Because of deviations of normality of the distributions of study variables, we used non-parametric tests (Mann-Whitney *U*-tests and Spearman's correlations).

In the multivariate analyses we investigated the relationships between RD, comorbid psychopathology, and QoL as dependent on gender. We tested moderated mediational models (Hayes, 2013) in which comorbid psychopathology, as measured by SDQ Difficulties scales, mediated the relationship between group membership (RD = 1, control = 0) and self- and parent-rated QoL, which was dependent on gender (boys = 0, girls = 1). Logarithmic transformations were used to assure normality. Bootstrapping with a resample procedure of 1000 bootstrap samples (bias corrected and accelerated (BCa) estimates and 95% CI) was used for significance testing, because this method does not impose the assumption of normality of the sampling distribution (Preacher and Hayes, 2008). Child's age and parents' level of education were included as covariates.

## Results

### Sample

Two hundred and eight children and adolescents were included in the study. Mean age of participants was 10.23 years (*SD* = 2.13, range: 6–15 years) in the RD group (*N* = 127) and 9.68 years (*SD* = 2.13, range: 7–15 years) in the control group (*N* = 81). The two groups did not differ significantly in age [*t*_(206)_ = 1.808, *p* = 0.072]. Demographic characteristics of the study groups are presented in Table 1.

**Table 1:** **Demographic characteristics of the study groups**.

	***N* (%)**		
	**RD (*n* = 127)**	**Control (*n* = 81)**	**χ^2^ (*p*)**
Gender			0.002 (0.964)
Girls	49 (38.6)	31 (38.3)	
Boys	78 (61.4)	50 (61.7)	
Father's level of education			5.847 (0.054)
Low	72 (56.7)	32 (39.5)	
Medium	39 (30.7)	35 (43.2)	
High	16 (12.6)	14 (17.3)	
No data available	0 (0)	0 (0)	
Father's economic activity			0.363 (0.547)
Economically active	99 (78.0)	64 (79.0)	
Other	27 (21.2)	14 (17.7)	
No data	1 (0.8)	3 (3.7)	
Mother's level of education			4.857 (0.088)
Low	55 (43.3)	23 (28.4)	
Medium	55 (43.3)	46 (56.8)	
High	17 (13.4)	12 (14.8)	
No data available	0 (0)	0 (0)	
Mother's economic activity			0.003 (0.956)
Economically active	91 (71.7)	59 (72.8)	
Other	33 (25.9)	21 (26.0)	
No data	3 (2.4)	1 (1.2)	
Family structure			0.009 (0.924)
Original full family	86 (67.7)	54 (66.7)	
Other	41 (32.3)	25 (30.8)	
No data available	0 (0)	2 (2.5)	

*N = 208. RD, Reading Disability*.

### Differences in QoL between the RD and control groups

Descriptive statistics and intercorrelations of study variables are presented in Table 2. Self- and parent-reported QoL ratings were significantly and positively related; the effect size was at a medium level, however. We found significant positive correlations of medium effect sizes among child's age and both self- and parent-reported ILK scores, indicating lower levels of QoL with increasing age. SDQ scores were not related to age. High levels of psychopathology were associated with low levels of QoL according to both self- and parent-reports (Table 2).

**Table 2:** **Descriptive statistics, reliabilities, and bivariate relationships (Spearman's rho) of study variables**.

	**Mean (*SD*)**	**Skew**	**Median (interquartile range)**	**ILK self-rated**	**ILK parent-rated**	**SDQ total difficulties**
Age	10.01 (2.15)			0.332^*^	0.337^*^	0.078
ILK Self-rated	13.61 (4.07)	0.679	13 (11–17)		0.327^*^	0.359^*^
IKL Parent-rated	13.31 (4.16)	0.865	12 (10–15)			0.429^*^
SDQ Total Difficulties	14.42 (5.56)	−0.201	15 (10–18)			

*N = 208. SD, Standard deviation; ILK, Inventar zur Erfassung Lebensqualität von Kindren und Jugendlichen; SDQ, Strength and Difficulties Questionnaire*.

* *p < 0.01*.

In the total sample, after a Bonferroni correction (α′ = 0.05/7 = 0.007), the RD group showed higher ILK scores in the school (*U* = 3674.0, *p* < 0.001), family (*U* = 3659.5, *p* < 0.001), time spent alone (*U* = 3972.5, *p* = 0.003), and mental health (*U* = 3472.5, *p* < 0.001) domains and general QoL (*U* = 3662.5, *p* < 0.001), according to the parent-reports, while no differences between RD and control groups were found, according to the self-reports. According to parent reports, the ILK total score was also higher in the RD group than in the control group (*U* = 3375.5, *p* < 0.001), but no differences were found between study groups, according to self-reports.

For boys, the RD group showed higher ILK scores in the family (*U* = 1299.0, *p* < 0.001) and mental health (*U* = 1127.0, *p* < 0.001) domains regarding general QoL (*U* = 1165.5, *p* < 0.001) as well as ILK total scores (*U* = 1155.0, *p* < 0.001), according to parent-reports, and again, no differences between the two groups were found, according to self-reports.

For girls, the RD group showed higher ILK score in the school domain (*U* = 486.5, *p* = 0.004), according to parent ratings, but no differences were found between the two groups, according to self-reports.

In the total sample, the two study groups did not differ in the total SDQ difficulties score. For boys, the RD group showed significantly higher scores in SDQ's total difficulties score than controls (*U* = 1407.5, *p* = 0.007), while in girls, no differences were found.

### The mediational role of psychopathology as dependent on gender

**Figure 1:**
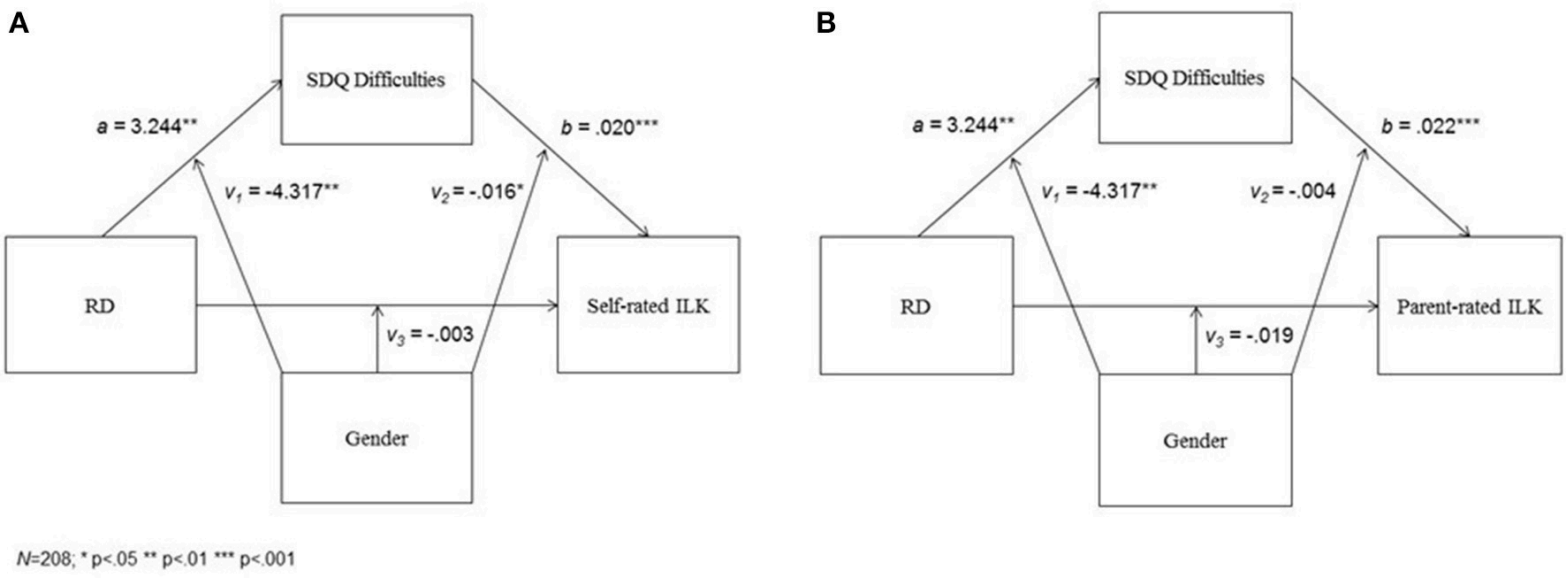
**The mediational effect of comorbid psychopathology in the relationship between group membership and QoL according to self- (A)** and parent- **(B)** ratings, as dependent from gender.

Results of the conditional process analyses are presented in Table 3 and Figures 1A,B.

**Table 3:** **Results of the conditional process analysis**.

	***B***	***SE***	***t***	***p***
**DEPENDENT: SDQ TOTAL DIFFICULTIES SCORE (MODEL 1 A AND B)**				
Intercept	9.605	1.891	5.081	<0.001
Child's age	0.187	0.077	1.052	0.294
Mother's level of education (= medium, ref = low)	−0.007	0.829	−0.009	0.993
Mother's level of education (= high, ref = low)	−2.254	1.152	−1.1956	0.052
Father's level of education (= medium, ref = low)	1.530	0.840	1.821	0.070
Father's level of education (= high, ref = low)	−2.314	1.163	−1.990	0.048
Child's gender (= girls, ref = boys)	4.856	1.199	4.051	<0.001
Group (= RD, ref = control)	3.244	0.958	3.384	0.001
Child's gender × Group	−4.317	1.543	−2.798	0.006
Model	*R*^2^ = 0.148, *F*_(8, 199)_ = 4.316, *p <* 0.001			
**DEPENDENT: ILK SELF-RATED (MODEL 1/A)**				
Intercept	1.958	0.095	20.655	<0.001
Child's age	0.044	0.008	5.381	<0.001
Mother's level of education (= medium, ref = low)	0.023	0.038	0.6200	0.536
Mother's level of education (= high, ref = low)	−0.111	0.053	−2.114	0.036
Father's level of education (= medium, ref = low)	−0.023	0.038	−0.610	0.542
Father's level of education (= high, ref = low)	0.066	0.053	0.113	0.910
Child's gender (= girls, ref = boys)	0.267	0.123	2.161	0.032
Group (= RD, ref = control)	−0.046	0.045	−1.029	0.305
SDQ Total difficulties score	0.020	0.004	5.338	<0.001
Child's gender × Group	−0.003	0.071	−0.047	0.962
Child's gender × SDQ Total difficulties score	−0.016	0.007	−2.170	0.031
Model	*R*^2^ = 0.280, *F*_(10, 197)_ = 7.646, *p* < 0.001			
**DEPENDENT: ILK PARENT-RATED (MODEL 1/B)**				
Intercept	1.905	0.091	20.865	<0.001
Child's age	0.036	0.008	4.567	<0.001
Mother's level of education (= medium, ref = low)	0.031	0.036	0.859	0.391
Mother's level of education (= high, ref = low)	−0.054	0.051	−1.063	0.289
Father's level of education (= medium, ref = low)	−0.041	0.037	−1.111	0.268
Father's level of education (= high, ref = low)	−0.042	0.051	−0.816	0.416
Child's gender (= girls, ref = boys)	0.053	0.119	0.448	0.655
Group (= RD, ref = control)	0.098	0.043	2.259	0.025
SDQ Total difficulties score	0.022	0.004	6.282	<0.001
Child's gender × Group	−0.019	0.069	−0.1278	0.781
Child's gender × SDQ Total difficulties score	−0.004	0.007	−0.527	0.599
Model	*R*^2^ = 0.360, *F*_(10, 197)_ = 11.090, *p* < 0.001			

*N = 208. B, unstandardized regression coefficients; SE, standard error of the unstandardized regression coefficient; R^2^, coefficient of determination; p, significance level; t and F, test-statistics; ILK, Inventar zur Erfassung Lebensqualität von Kindren und Jugendlichen; RD, Reading Deficit; SDQ, Strength and Difficulties Questionnaire*.

**Figure 2:**
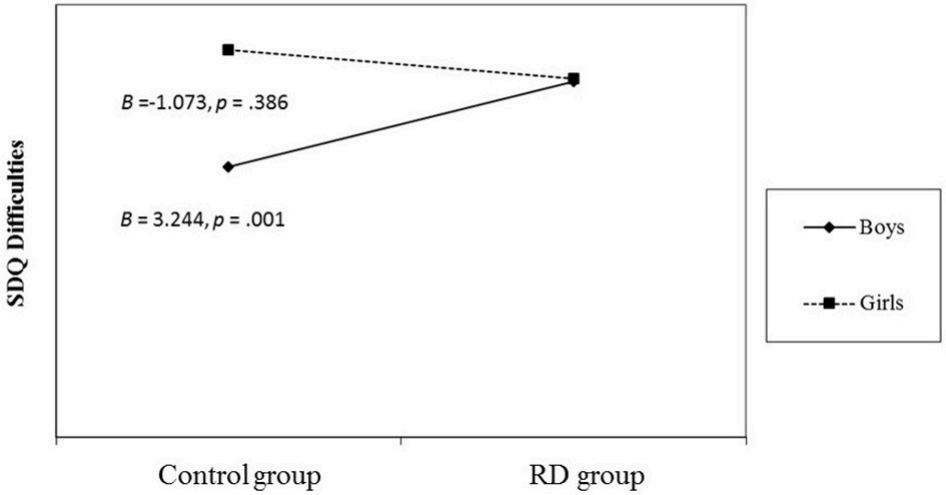
**Regression lines for relations between group membership and psychopathology as moderated by gender (a 2-way interaction)**. *B* = unstandardized regression coefficient (i.e., simple slope). RD, Reading Deficit; SDQ, Strength and Difficulties Questionnaire.

Group status and gender, as well as the interaction term of these variables were significantly related to SDQ scores, when controlling for child's age and parents' level of education. A *post-hoc* analysis (Holmbeck, 2002) revealed that for boys, having an RD diagnosis was associated with higher SDQ scores, while in girls SDQ scores did not differ between the RD and control groups (Figure 2).

**Figure 3:**
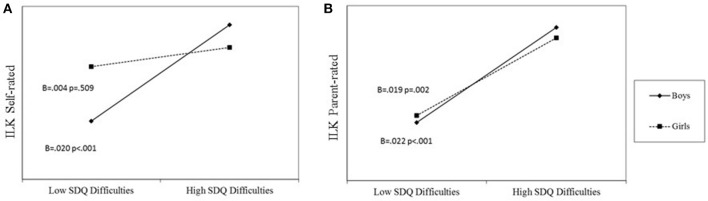
**Regression lines for relations between psychopathology and QoL according to self- (A)** and parent- **(B)** ratings, as moderated by gender (a 2-way interaction).

In the first model, presented in Figure 1A, self-rated ILK scores were related to SDQ total difficulties score, gender, and the interaction term of these variables. Group membership and the interaction term of group membership by gender were not associated with self-reported ILK scores, again, when controlling for child's age and parents' level of education. The *post-hoc* analysis revealed that, for boys, higher SDQ scores were related to higher ILK scores, but no relationship was found for girls (Figure 3A).

The conditional direct effects of group membership on self-rated ILK scores were non-significant for both boys (= −0.046, BCa 95% CI: −0.135 to 0.043) and girls (= −0.050, BCa 95% CI: −0.161 to 0.061). The conditional indirect effect of group membership on self-rated ILK scores through SDQ total difficulties score was significant for boys (= 0.064, BCa 95% CI: 0.024–0.111), but non-significant for girls (= −0.004, BCa 95% CI: −0.041 to 0.008), and the difference between the conditional indirect effects was significant (= −0.068, BCa 95% CI: −0.120 to −0.022).

In the second model, presented in Figure 1B, parent-rated ILK scores were related to SDQ total difficulties score and group status. Gender and the interaction terms of group membership by gender as well as SDQ scores by gender were not associated with parent-reported ILK scores, again, when controlling for child's age and parents' level of education. For both boys and girls, higher SDQ scores were related to higher ILK scores (Figure 3B).

The conditional direct effect of group membership on parent-rated ILK scores was significant for boys (= −0.098, BCa 95% CI: 0.012–0.184), but not for girls (= 0.079, BCa 95% CI: −0.028 to 0.186). The conditional indirect effect of group membership on self-rated ILK scores through SDQ total difficulties score was significant for boys (= 0.073, BCa 95% CI: 0.029–0.127), but non-significant for girls (= −0.020, BCa 95% CI:−073 to 0.013), and the difference between the conditional indirect effects was significant (= −0.093, BCa 95% CI: −0.164 to −0.034).

## Discussion

Our study extends several aspects of the existing, but very limited data on the QoL of children with an RD (Karande et al., 2009; Karande and Bhosrekar, 2009; Rotsika et al., 2011; Karande and Venkataraman, 2012, 2013; Ginieri-Coccossis et al., 2013).

Our first hypothesis was supported. We measured the QoL of the children from both RD and healthy control groups, and we supported the data from population and clinical studies (Bastiaansen et al., 2005; Michel et al., 2009): QoL decreases with increasing age in childhood, according to both self- and parent-reports. During adolescence, children experience a transition period, as they face several changes, including psychical (e.g., hormonal) changes, new social roles (e.g., being less dependent on parents yet still living at home, finding their place among peers, having their first girlfriends or boyfriends), and experiencing psychological changes; they need time and good coping mechanism to adapt (Plancherel and Bolognini, 1995; Hampel, 2007; Patton and Viner, 2007; Michel et al., 2009). The tension of these changes can lead to lower QoL.

Our second hypothesis was supported. Similar to the study of Bastiaansen et al. (2005), who investigated children in a general psychiatric outpatient clinic, a higher level of psychopathology was associated with lower QoL in our study population, when we included both RD and healthy control groups and boys and girls together, again, based on both self- and parent-reports. We agree with the suggested explanations of Bastiaansen et al. (2005) as older children are already more aware of their difficulties due to their psychopathology than younger children, and, as they become older, the duration of their psychopathology can be longer, which has greater impact on their QoL.

Our third hypothesis was only partly supported. When we compared the QoL of the RD and the control groups, based on both self- and parent-reports, the parents in the RD group rated several aspects (i.e., school, family, mental health, and general QoL) of their children's QoL as worse than parents rated the QoL of their control group children and the children with an RD did not rate their QoL lower in the above mentioned domains as their healthy pears did. It is very interesting when we measured gender differences that we got the same result for boys; however, in the case of girls, not only self-reported, but parents-reported QoL of children with an RD did not differ from the control group, except in the school domain. Our results, especially in the case of boys, belong to the line of those studies, which highlight that there is a discrepancy between self- and parent-rated QoL of children (Eiser and Jenney, 2007; Danckaerts et al., 2009; Eiser and Varni, 2013). It is not so surprising that parents of boys with RD rated their children's QoL lower than did the parents of healthy boys because they know all the difficulties their children experience due to their RD. This result for boys even supports those very few previous studies on children with SpLD, where parents reported about their children QoL (Karande et al., 2009). However, it is interesting why parents of girls with RD did not rate their children's QoL lower in many domains, except school, than did the parents of healthy girls. Below, we will give some possible explanation of this. Our results are partly similar to the findings of Ginieri-Coccossis et al. (2013); according to self-report, no differences were found between RD and control groups for both genders in the QoL dimensions of physical well-being and school functioning. Moreover, the very few previous studies measuring the self-rated QoL of children with SpLD reported lower self-rated QoL of children with SpLD than typically developing children in the dimensions of emotional well-being and family and friends (Karande et al., 2009; Rotsika et al., 2011; Karande and Venkataraman, 2012; Ginieri-Coccossis et al., 2013). However, in our study, children (both girls and boys) did not rated their QoL lower on the dimension of family and general QoL than did healthy children. Our results raise the question of whether children in our population underestimate, deny, or do not experience the consequences of their RD. Or it can be, that as in Hungary there is a low level of stigma around SpLD and children with SpLD get state sponsored special educational development and easier tasks in the school, they do not experience negative consequences of SpLD o their QoL. Further studies are needed to clarify this question.

Our fourth hypothesis was again only partly supported. In our study, psychopathology was rated by the parents. Surprisingly, when we compared the RD and the control group, we did not find differences in the level of psychopathology; however, when we examined girls and boys separately, as we expected, parents of boys in the RD group reported more psychopathology than parents in the control group, but the parents of girls with RD did not report more psychopathology than the parents of healthy girls. Later, we will come back to this result.

Our study is the third one to our knowledge that has investigated the role of comorbidity on the QoL of children with RD; moreover, none of the previous studies investigated gender differences within this topic (Karande and Bhosrekar, 2009; Karande and Venkataraman, 2013). Our fifth hypothesis was again only partly supported. We were interested in the relationships between age and gender as well as RD and comorbid psychopathology and QoL. The most notable are the gender differences: while boys' comorbid psychopathology mediated between RD and both self- and parent-rated QoL; moreover, we found a direct association between boys with RD and their parent-rated QoL. In the case of girls, neither direct nor indirect effects of comorbid psychopathology were found between RD and QoL, based on both self- and parent-reports.

Further investigating this gender differences, we found higher levels of psychopathology in girls than in boys within the control group; moreover, even control group girls with lower (i.e., subclinical) levels of psychopathology rated their QoL poorer than boys did. All these can explain the surprising result described above: in the case of girls, we did not find differences between the QoL of those with an RD and healthy children, based not only on self- but on parent-reports as well. Parents of girls in both groups reported higher levels of psychopathology, but they did not rate their daughters' QoL lower, while the parents of boys did, as we described above. These results highlight very important gender differences in the population in this age group. Knowing that externalizing psychopathology is usually more disturbing for the surrounding family, teachers and pears (Klassen et al., 2006), an explanation of the lower parent-rated QoL of boys could be that boys may have more externalizing comorbidity than girls in our study, but we did not examine this question.

Moreover, these data support those previous studies that reported on the importance of subclinical/subthreshold psychopathology even in children (e.g., Lewinsohn et al., 2004; Klein et al., 2009; Bussing et al., 2010; Bertha and Balázs, 2013; Balázs and Keresztény, 2014). Subthreshold psychiatric disorders are defined as psychiatric conditions that do not fulfill the criteria of the classification systems (i.e., the DSM-5; American Psychiatric Association, 2013) and International Classification of Diseases, 10th edition (ICD-10) (World Health Organization, 1992); however, the symptoms cause impairment and significant suffering for the child or adult and his or her environment, e.g., family, teachers, colleagues, friends. In our study, the girls with subthreshold psychopathology, who reported poorer QoL, exactly reflects this issue and highlights the importance of screening already subthreshold symptoms, especially in girls, to prevent poorer QoL.

Finally, as we expected, high levels of psychopathology was associated with low parent-rated QoL in the cases of boys and girls. It draws the attention of clinicians to the importance of recognizing and treating comorbidity in conjunction with an RD.

Several limitations should be considered when interpreting our findings. First of all, it is a cross-sectional study, which prevents us from making causal attributions. Second, data on psychopathology analyzed in this study rely only on parent-reported information. This could be a source of bias. Third, we did not examine if the children had externalizing or internalizing comorbid psychopathology. Externalizing and internalizing psychopathology could have different effects on QoL. Fourth, self-rated data on QoL could be biased as well, while when data refer to personally sensitive information, persons tend to give socially acceptable answers.

In conclusion, the results of this study indicate that QoL is a useful measure of well-being in the case of children with an RD, and they highlight the importance of measuring comorbid psychopathology, even on a subthreshold level and accounting for gender and age differences. Moreover, we would like to underline the importance of getting information on psychopathology and QoL from more sources.

## Author contributions

JB ideated and designed the study, trained the personnel and jointly drafted the manuscript. MM made the statistical analyses and jointly drafted the manuscript. KT coordinated the implementation, trained the personnel and jointly drafted the manuscript. DN participated in the data collection and in the statistical analyses and jointly drafted the manuscript.

## Funding

This work was partly supported by OTKA K108336 grant. JB was supported by the János Bolyai Research Scholarship of the Hungarian Academy of Sciences.

### Conflict of interest statement

JB has received a speaker honorarium from E. Lilly Company and she is a member of the Advisory Board committee of E. Lilly Company. The other authors declare that the research was conducted in the absence of any commercial or financial relationships that could be construed as a potential conflict of interest.
